# Irreversible repolarization of tumour‐associated macrophages by low‐Pi stress inhibits the progression of hepatocellular carcinoma

**DOI:** 10.1111/jcmm.17861

**Published:** 2023-07-20

**Authors:** Yang‐feng Lv, Zi‐qiang Liao, Qiu‐chen Bi, Chuan‐sheng Xie, Xiao‐yong Wei, Yi Yun, Yuan‐qiao He, Qun Tang

**Affiliations:** ^1^ Jiangxi Provincial Key Laboratory of Preventive Medicine, School of Public Health Nanchang University Nanchang China; ^2^ Institute for Advanced Study, Nanchang University Nanchang China; ^3^ Department of Hepatobiliary Surgery Jiangxi Provincial Cancer Hospital Nanchang China; ^4^ Biobank Center The Second Affiliated Hospital of Nanchang University Nanchang China; ^5^ Center of Laboratory Animal Science, Jiangxi Province Key Laboratory of Laboratory Animal Nanchang University Nanchang China

**Keywords:** hepatocellular carcinoma, low‐Pi stress, macrophage polarization, tumour‐associated macrophages

## Abstract

Numerous studies have shown the positive correlation between high levels of Pi and tumour progression. A critical goal of macrophage‐based cancer therapeutics is to reduce anti‐inflammatory macrophages (M2) and increase proinflammatory antitumour macrophages (M1). This study aimed to investigate the relationship between macrophage polarization and low‐Pi stress. First, the spatial populations of M2 and M1 macrophages in 22 HCC patient specimens were quantified and correlated with the local Pi concentration. The levels of M2 and M1 macrophage markers expressed in the peritumour area were higher than the intratumour levels, and the expression of M2 markers was positively correlated with Pi concentration. Next, monocytes differentiated from THP‐1 cells were polarized against different Pi concentrations to investigate the activation or silencing of the expression of p65, IκB‐α and STAT3 as well as their phosphorylation. Results showed that low‐Pi stress irreversibly repolarizes tumour‐associated macrophages (TAMs) towards the M1 phenotype by silencing stat6 and activating p65. Moreover, HepG‐2 and SMCC‐7721 cells were cultured in conditioned medium to investigate the innate anticancer immune effects on tumour progression. Both cancer cell lines showed reduced proliferation, migration and invasion, as epithelial–mesenchymal transition (EMT) was inactivated. In vivo therapeutic effect on the innate and adaptive immune processes was validated in a subcutaneous liver cancer model by the intratumoural injection of sevelamer. Tumour growth was significantly inhibited by the partial deprivation of intratumoural Pi as the tumour microenvironment under low‐Pi stress is more immunostimulatory. The anticancer immune response, activated by low‐Pi stress, suggests a new macrophage‐based immunotherapeutic modality.

## 1 BACKGROUND

Hepatocellular carcinoma (HCC) is the fifth most commonly diagnosed malignancy and the second most common cause of death from cancer worldwide. Although some breakthroughs have been made in terms of diagnostic and treatment capabilities, the prognosis of hepatocellular carcinoma is still frustrating due to high postsurgical recurrence and a high metastatic rate (50%–70% over 5 years).^1^ Chronic inflammation is the predominant risk factor for inducing HCC.^2^ Further investigation indicates that tumour‐associated immune cells in the microenvironment are closely associated with HCC progression, and immunotherapy targeting those cells has shown great potential.^3^, ^4^, ^5^, ^6^ Clinical studies and experimental mouse models suggest that tumour‐associated macrophages (TAMs) are particularly abundant among innate and adaptive immune cells recruited to the tumour environment.^7^, ^8^ TAMs are classified as M1 (classically activated) or M2 (activated). M2 macrophages represent a significant proportion of TAMs. Due to their plasticity, the two polarization forms can be reprogrammed to each other in the presence of defined stimuli.^9^

Many cytokines are involved in the polarization of M2 macrophages,^10^ and M2 macrophages can secrete several complex immunosuppressive factors, cytokines, matrix metalloproteases and growth factors. Therefore, they can regulate the Th2‐type immune response, promote tumour cell growth and participate in tumour angiogenesis.^11^, ^12^ By contrast, M1 macrophages are activated by stimuli including LPS, TNF‐α, GM‐CSF, IFN‐γ and Toll‐like receptor (TLR) ligands.^13^ M1 macrophages secrete proinflammatory cytokines and chemokines, such as TNF‐α, IL‐6, IL‐12, CCL4 and CXCL10. In addition, M1 macrophages exhibit high expression of major histocompatibility complex type II (MHC‐II), which can regulate and promote the Th1‐type cellular immune response by presenting antigens to T cells. M1 macrophages are essential tumour‐suppressing cells that initially act in the tumour microenvironment.^14^

The population of M2 phenotypes is correlated with poor prognosis in numerous malignancies, and an increased M1/M2 TAM ratio has been associated with an improved 5‐year prognosis in some solid tumours, including HCC.^15^, ^16^ Thus, a central goal of macrophage‐based cancer therapeutics is, stated simply, to reduce anti‐inflammatory macrophages and increase proinflammatory (antitumour) macrophages.^17^ Past years have witnessed the modulation of macrophage repolarization for cancer therapy. Huang et al found that chloroquine modulates the antitumour immune response by resetting tumour‐associated macrophages towards the M1 phenotype.^18^ Astragaloside IV inhibits lung cancer progression and metastasis by modulating macrophage polarization through AMPK signalling.^19^ Recently, Michal et al. demonstrated that targeting cancer glycosylation repolarizes TAMs from the M2 to M1 phenotype, allowing effective immune checkpoint blockade.^20^

The collected evidence suggests that Pi, as a signalling molecule, participates in the progression and metastasis of cancer.^21^ M S. Razzaque et al. noted that under the stress of excessive Pi burden, tumour progression can be triggered by accelerating angiogenesis, inducing chromosome instability, and promoting metastasis.^22^ In 2007, Valery V. Khramtsov et al. observed that there are 2‐fold higher concentrations of interstitial Pi in tumours than in normal tissues.^23^ More importantly, Villa‐Bellosta R. et al. found that phosphates can prevent ectopic calcification by mediating macrophage polarization.^24^ Herein, we try to elucidate the relationship between Pi starvation‐induced macrophage polarization and the immune anticancer effect in vitro and in vivo, expecting low‐Pi stress will become a new immunotherapy to be used as a single protocol or in combination with other treatments.

In this study, we correlated the spatial Pi concentration with the biomarker of the M1/M2 phenotype from 22 HCC specimens. In vitro tests confirmed that low‐Pi stress modulated the balance between M1 and M2 macrophages towards a more proinflammatory status, shifting to a higher M1/M2 ratio in vitro, and repolarization of macrophages suppressed tumour growth in a murine liver cancer model. The mechanism of low‐Pi‐induced macrophage polarization was fully explored. We also determined that polarized macrophages inhibit tumour progression via both innate immunity and adaptive immunity.

## 2 MATERIALS AND METHODS

### 2.1 Reagents

Sevelamer, IL‐4, Phorbol 12‐myristate13‐acetate (PMA), LPS and IFN‐γ were purchased from MCE® (Shanghai, China); antibodies were acquired from different manufacturers: rabbit antihuman CD206 polyclonal antibody (TD4149S;1:1000), rabbit antihuman iNOS polyclonal antibody (TA0199;1:1000), rabbit antihuman Stat3 polyclonal antibody (T55292S;1:1000), Rabbit antihuman p‐Stat3 polyclonal antibody (T56566S;1:500), rabbit antihuman Stat6 polyclonal antibody (T55351S;1:2000), rabbit antihuman p‐Stat6 polyclonal antibody (TA3301S;1:1000), rabbit antihuman p65 polyclonal antibody (T55034S;1:2000), rabbit antihuman p‐p65 polyclonal antibody (TA2006S;1:1000), rabbit antihuman IкBα polyclonal antibody (T55026F;1:2000), rabbit antihuman p‐IкBα polyclonal antibody (TP56280F;1:1000), rabbit antihuman E‐cadherin polyclonal antibody (TA0131S;1:1000), rabbit antihuman vimentin polyclonal antibody (T55134S;1:1000), rabbit antihuman snail polyclonal antibody (PA2086S;1:1000), rabbit antihuman fibronectin polyclonal antibody (TA5335S;1:2000) and rabbit antihuman β‐actin monoclonal antibody (P30002S;1:5000) were purchased from Abmart® (Shanghai, China); rabbit antihuman AKT monoclonal antibody (60203‐2‐Ig;1:5000), rabbit antihuman p‐AKT recombinant antibody (80455‐1‐RR;1:5000) and rabbit antihuman PCNA monoclonal antibody (60097‐1‐Ig;1:2000) were purchased from Proteintech® (Wuhan, China); APC anti‐Mouse CD206 antibody (E‐AB‐F1135E) and PE anti‐Mouse CD86 antibody (E‐AB‐F1012D) were obtained from Elabscience (Wuhan, China).

### 2.2 Patient samples

All HCC patient samples, including intratumour and peritumoural liver tissues, were obtained from the Second Affiliated Hospital of Nanchang University from September 2021 to September 2022. The present study was approved by the Ethics Committee of the Second Affiliated Hospital of Nanchang University (NO. 2021–12). Half of the collected tissues were fully ground until all the cells and cell organs were fragmented at 4°C for Pi content determination, and the remaining tissues were ground in liquid nitrogen for Q‐PCR detection.

### 2.3 Determination of Pi content

Tissues, including intratumour tissues and peritumour tissues in the patient sample or the mouse tumour tissues, were placed in saline (1 mL), and a tissue homogenizer was used for homogenization. After centrifugation (12,000 rpm, 10 min), the supernatant was collected, and the mass concentration of inorganic phosphate (Pi) in each independent sample was detected by phosphomolybdic acid colorimetry.^25^, ^26^ Specifically, an appropriate volume of the sample to be tested is added to a 1.5 mL centrifuge tube. Then, 200 μL of different concentrations of the standard phosphate solution and sample to be tested are sequentially pipetted into the sample wells of a 96‐well plate. 70 μL of colour reagent is added to each well, and the mixture is incubated at room temperature for 30 min. The absorbance at 630 nm is measured using a microplate reader. The phosphate concentration of the sample is calculated based on the standard curve and the dilution factor of the sample. Each sample group is tested with three technical replicates.

### 2.4 Animal model

Six‐ to eight‐week‐old C57 mice were trained adaptively for 7 days before the experiments. Mice were injected subcutaneously with 1 × 10^6^ Hepa1‐6 cells in 100 μL PBS. After 14 days, the mice were divided into two groups (*n* = 5): the control group mice were injected with normal saline in the tumour, and the treatment group mice were injected with 1 mg sevelamer every day in 100 μL of normal saline for 14 days. The weight of the tumour was evaluated, and the tumour volumes were calculated by the following formula: tumour volume = (longest diameter) × (shortest diameter)^2^/2.

### 2.5 Cell lines and cell culture

The human monocyte cell line THP‐1 and HCC cell lines (HepG‐2 and SMMC‐7721) were purchased from the Cell Bank of the China Science Academy (Shanghai, China). Here, THP‐1 cells, HepG‐2 cells and SMMC‐7721 cells were cultured in RPMI‐1640 medium, and all cultures were supplemented with 10% FBS and incubated with 5% CO_2_ at 37°C.

### 2.6 Construction of the macrophage polarization model

THP‐1 cells were cultured in 320 nmoL/L PMA for 18 h to induce differentiation into M0 macrophages. The M0 macrophages were then polarized to M1 macrophages by treatment with 100 nmoL/L PMA plus 100 ng/mL LPS and 20 ng/mL IFN‐γ for 48 h and M2 macrophages by incubation with 100 nmoL/L PMA supplemented with 20 ng/mL IL‐4 for 48 h.

### 2.7 Pi deprivation and recovery culture of macrophages

The Pi‐deprived medium was prepared by adding the corresponding quality of sevelamer to the RPMI‐1640 medium, where different Pi concentration gradients (800, 600, 400, 200, 100, 50 and 0 mg/L) were used. According to the instructions provided by the manufacturer, it is known the Pi concentration of normal RMPI‐1640 culture medium is 800 mg/L, and the phosphoric acid binding rate of sevelamer is 5.5 mmoL/g. Add sevelamer reaction for 4 h, the medium was filtered using 0.22 μm filter membrane, and two functions are highlighted here: filter out bacteria and sevelamer combined with phosphate. It is worth noting that sevelamer is not soluble in cell culture medium.

For Pi recovery assays, after Pi deprivation of cells, the corresponding volume of phosphate buffer solution (PBS) was added to the original culture medium to restore the Pi concentration in the medium to the normal level (800 mg/L), continue to cultivate for 12 h or 24 h.

### 2.8 Conditioned medium (CM) preparation

When THP‐1 cells were polarized into different models (M0‐type, M1‐type and M2‐type), these macrophages were cultured in serum‐free medium for 24 h, and the supernatant was collected by centrifugation (12,000 rpm, 10 min) and stored at −80°C.

### 2.9 MTT assays

The viability of cells with different polarization states exposed to media with different concentrations of Pi was evaluated using the MTT assay. The MTT assay was also used to examine the effect of the conditioned medium on the cell viability of HepG‐2 and SMCC‐7721 cells. In detail, the cells were plated on 96‐well plates at a density of 1*10^6^ cells/mL. After 12 h, the medium was removed and changed to a special medium. In brief, medium of different Pi concentrations (800, 600, 400, 200, 100, 50 and 0 mg/L) was added to M0‐type, M1‐type and M2‐type macrophages for 48 h, and HepG‐2 and SMCC‐7721 cells were changed to conditioned medium (M0‐CM, M0 + Pi(−)‐CM, M2‐CM, M2 + Pi(−)‐CM, M1‐CM and M1 + Pi(−)‐CM) for 48 h. After the specific culture, we added 10 μL of MTT reagent to each well and then cultured the cells for 4 h. After the supernatant in the 96‐well plate was discarded, 100 μL of dimethyl sulfoxide (DMSO) was added to each well and agitated on a horizontal shaker for 10 min. Finally, the OD value of each well was detected at 490 nm using a microplate reader (Thermo Fisher Scientific, USA). Cell viability was calculated as follows: (OD _Treated group_—OD _Blank group_)/(OD _Control group_—OD _Blank group_) × 100%.

### 2.10 Flow cytometry analysis

Flow cytometry was used to detect the immunophenotype of macrophages and the apoptosis of HepG‐2 cells in the conditioned medium. First, for apoptosis detection, HepG‐2 cells were cultured in conditioned medium for 24 h and later collected. The cells were washed twice with ice‐cold PBS, resuspended in 400 μL of binding buffer, and incubated with Annexin V (10 μL) and PI labelling solution (5 μL) at room temperature for 10 min. Subsequently, the percentage of apoptotic cells was measured using a flow cytometer.

To detect the immunophenotype of macrophages, macrophages were processed into single‐cell suspensions and incubated with antibodies (APC‐CD206, PE‐CD86) for 1 h at 4°C. The cells were then washed twice with 4 mL of flow buffer, centrifuged and resuspended in 0.3 mL of flow buffer for analysis. Flow cytometry was performed using a FACSCalibur flow cytometer (BD Biosciences, USA). Flow cytometric analysis was performed using FlowJo software (FlowJo, USA).

### 2.11 Real‐time PCR (RT–PCR)

Total RNA was isolated from cells or tissues using an RNA extraction kit (TIANGEN, Beijing), and a total of 1 μg cDNA was synthesized using the Prime Script RT Reagent Kit (Takara). The sequences of the primers used for RT–PCR are listed in Table 1. Quantitative real‐time RT–PCR (Q‐PCR) was performed in Eight pipe using Takara SYBR Green reagents. All processes were carried out in strict accordance with the manufacturer's instructions.

**TABLE 1:** Primers used for the amplification of human gene and mouse gene.

Name	Sequences(5′‐3′)
H‐β‐Actin‐F	TGGACTTCGAGCAAGAGATG
H‐β‐Actin‐R	GGATGTCCACGTCACACTTC
H‐CD68‐F	GGAAATGCCACGGTTCATCCA
H‐CD68‐R	TGGGGTTCAGTACAGAGATGC
H‐CD206‐F	CTACTGTTATGTCGCTGGCAAA
H‐ CD206‐R	GGATGGAAGCAAAGTGGATTAG
H‐IL‐10‐F	GATCTCCGAGATGCCTTCAG
H‐IL‐10‐R	ATCGATGACAGCGCCGTAGC
H‐CCL17‐F	CCCTGCACAGTTACAAAAACGA
H‐CCL17‐R	GAGCCATTCCCCTTAGAAAGCT
H‐TGF‐β‐F	GAACCCGTTGATGTCCACTT
H‐TGF‐β‐R	CACGTGGAGCTGTACCAGAA
H‐iNOS‐F	CACGGCCTTGCTCTTGTTTT
H‐iNOS‐R	GTGATGCCCCAAGCTGAGA
H‐COX2‐F	TAAGTGCGATTGTACCCGGAC
H‐COX2‐R	TTTGTAGCCATAGTCAGCATTGT
H‐TNFα‐F	GGCCAGAGGGCTGATTAGAGA
H‐TNFα‐R	CTTCTGCCTGCTGCACTTTG
H‐CCR7‐F	GTAATCGTCCGTGACCTCATCTT
H‐CCR7‐R	GCTGGTGGTGGCTCTCCTT
M‐β‐Actin‐F	CCACCATGTACCCCAGGCATT
M‐β‐Actin‐R	AGGGTGTAAAACGCAGCTCA
M‐CD206‐F	GTTCACCTGGAGTGATGGTTCTC
M‐CD206‐R	AGGACATGCCAGGGTCACCTTT
M‐iNOS‐F	CAGCTGGGCTGTACAAACCTT
M‐iNOS‐R	CATTGGAAGTGAAGCGTTTCG

### 2.12 Western blot analysis

Western blotting was performed using a standard protocol. Briefly, the cells or tissues were harvested in RIPA lysis buffer contained 10% PMSF (Phenylmethylsulfonyl fluoride) and incubated for 30 min on ice. The supernatant was collected after centrifugation (12,000 rpm) at 4°C for 10 min. The protein concentration was measured by a protein concentration determination kit (Beyotime Biotechnology). Approximately 10 μg of protein was electroblotted onto a PVDF membrane following electrophoretic separation on a 10% SDS‐polyacrylamide gel. Subsequently, the membranes were blocked with 5% nonfat milk at room temperature for 1 h and then incubated with specific primary antibodies overnight at 4°C. Blots were washed three times for 15 min with Tris‐Buffered Saline and 0.1% Tween 20 (TBST) and then incubated with a 1:1000 dilution of HRP‐conjugated secondary antibody for 2 h at room temperature. Ultimately, the membranes were again washed three times for 15 min with TBST and then visualized with enhanced chemiluminescence (ECL) reagent (Thermo Scientific, USA). All antibodies were diluted with 5% nonfat milk powder according to the manufacturer's instructions, and all Western blotting images for this study are representative of at least three independent biological experiments. Finally, the results were analysed using ImageJ software (USA).

### 2.13 Immunofluorescence staining

A total of 5 × 10^5^ cells were cultured in confocal dishes and treated with a special medium, including normal Pi (Control), Pi(−), IL‐4(M2)/LPS + IFN‐γ(M1) and IL‐4 + Pi(−)/LPS + IFN‐γ + Pi(−) treatment groups. A total of two arrays were executed here: the first array included two groups (CD206 and iNOS), and the second array included two groups (p65 and Stat6). After treatment, the cells were fixed with 4% paraformaldehyde for 30 min at room temperature (RT) and washed three times with 5% PBS‐Tween (PBST) for 15 min. Subsequently, the cells were blocked in 1% BSA at RT for 1 h and then incubated with specific primary antibodies at 4°C overnight. After washing three times with 5% PBST for 15 min, the cells were stained with an Alexa Fluor‐488 secondary antibody for 2 h at RT and then again washed three times with 5% PBST. Finally, the cells were counterstained with DAPI, and the cell images were captured using a confocal microscope.

For tissue samples, tumour tissues were removed and fixed in 4% paraformaldehyde at 4°C for 3 days, dehydrated in 30% sucrose and finally embedded in paraffin. Then, the sections were cut into 5 μm thick continuous sections, incubated with antirabbit CD206, iNOS, CD34, CD4 and CD8 antibodies, and stained with Alexa Fluor 594 conjugate secondary antibody (1:1000 dilution). All cells were visualized with a laser confocal scanning microscope (Olympus, Japan).

### 2.14 Wound healing assay

HepG‐2 cells or SMCC‐7721 cells were plated on 6‐well plates (5*10^5^ cells/well). When the cells were cultured for 12 h, a space without cells was obtained by a disposable sterile 10 μL pipette. The dropped cells were washed three times with PBS, and conditioned medium, including M0‐CM, M0 + Pi(−)‐CM, M2‐CM, M2 + Pi(−)‐CM, M1‐CM and M1 + Pi(−)‐CM, was added. Images of scratches in each group were captured using an inverted microscope. Subsequently, the cells were returned to the incubator for an additional 48 h. After culture termination, the image at the scratch was acquired again using an inverted microscope. Three random fields along the scraped line were selected and quantified with ImageJ software.

### 2.15 Cell migration and invasion analysis

2*10^5^ cells were cultured in inserts with 8 μm pores (Corning), which were inserted into 24‐well plates. After the cells adhered to the wall, the medium was discarded and changed to conditioned medium as previously described, and the cells were cultured for 48 h. The inserts were removed and washed three times with PBS. The cells were fixed with 4% paraformaldehyde, and invasion analysis was performed by staining with a crystal violet dye solution. The cell image at the bottom of the insert was captured after wiping the cells inside the insert with a cotton swab. Similar to migration, matrix glue was added at the bottom of the insert in the invasion experiment according to the manufacturer's instructions. The results were analysed using ImageJ software.

### 2.16 ELISA

The cell culture supernatant of each group was collected and centrifuged at 1000 × g for 20 min, and then the supernatant was transferred into new tubes, detected immediately, or stored in an aliquot at −80°C for later use. The amounts of IL‐10 and TNF‐α in the supernatant were detected by using the Human IL‐10 ELISA Kit (mlbio) and Human TNF‐α ELISA Kit (mlbio), respectively, according to the manufacturer's instructions. Briefly, 50 μL of standard or sample was added to the wells, and 100 μL of enzyme conjugate was added to the standard and sample wells, covered with an adhesive strip, and incubated for 60 min at 37°C, after which the plate was washed four times with wash solution. Fifty microliters of substrate A and 50 μL of substrate B were added to each well. The samples were gently mixed and incubated for 15 min at 37°C in a dark environment. Fifty microliters of stop solution was added to stop the reaction, and then the plate was read at 450 nm by using a microtiter plate reader within 15 min.

### 2.17 Morphological analysis and immunohistochemistry

The tumours collected from each group of mice were fixed with 4% paraformaldehyde, embedded in paraffin, and cut into 5 μm sections. Haematoxylin and eosin staining was performed and used to measure the tumour burden. Moreover, tumour sections were incubated with 3.0% hydrogen peroxide/oxide to inactivate endogenous peroxidase. After antigen repair, the sections were blocked with 5% bovine serum and incubated with primary antibodies (anti‐FOXP_3_ and anti‐PCNA) at 4°C. Subsequently, the sections were washed with PBS, further labelled with a biotin‐conjugated secondary antibody, stained with 3,3′‐diaminobenzidine (DAB) and counterstained with haematoxylin for observation on an inverted microscope (Lecia, Germany).

### 2.18 Statistical analysis

Data are expressed as the means ± standard errors of the means (SEM). The statistical significance of the differences was tested using one‐way anova or Student's *t* tests. A *p* value less than 0.05 was regarded as statistically significant. All statistical analyses were performed in GraphPad 9.0.

## 3 RESULTS

### 3.1 Correlation between macrophages and Pi content in the tumour microenvironment of HCC patients

**FIGURE 1:**
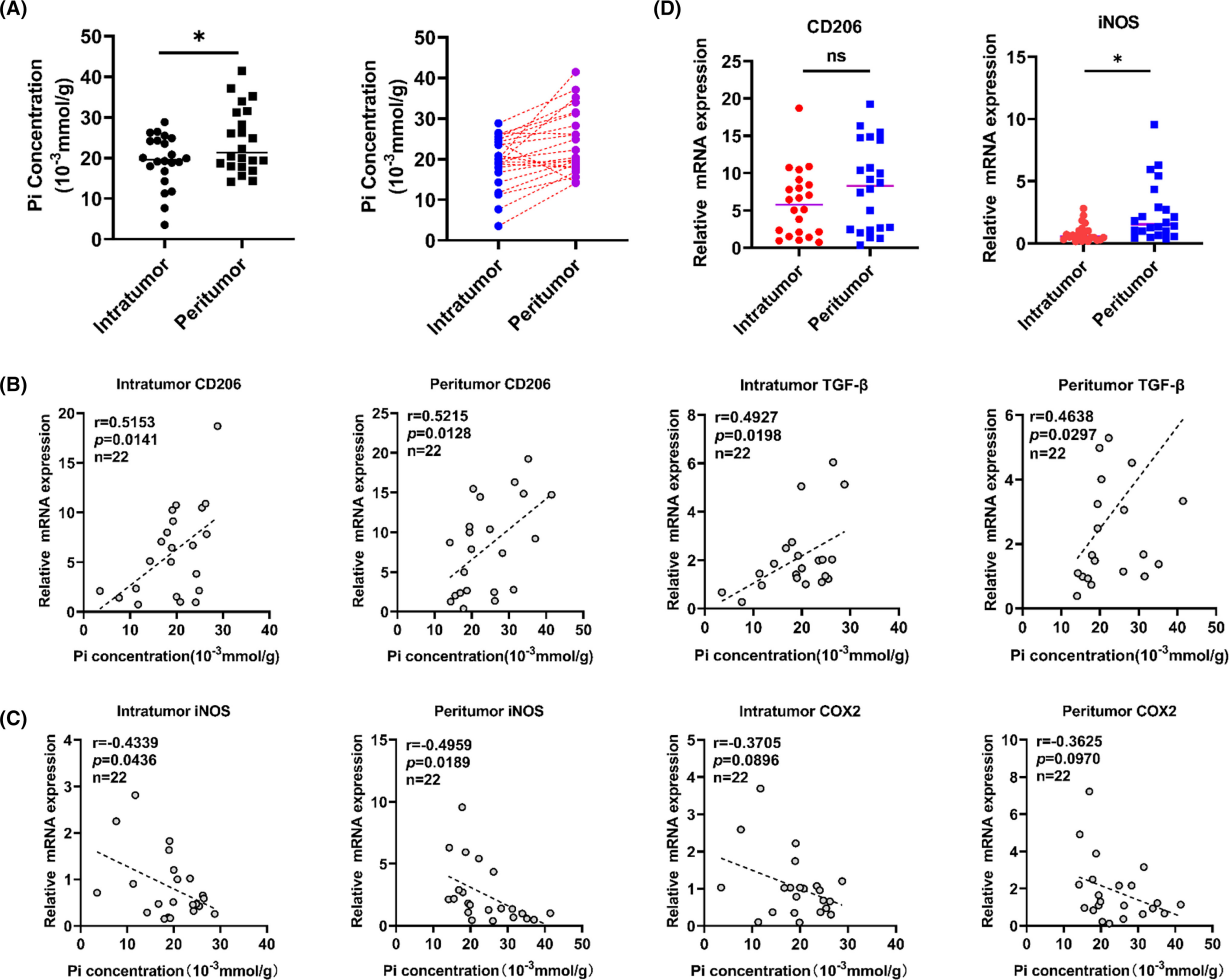
Pi content and transcript analyses of macrophages in clinical HCC patient samples. Both intratumoural and peritumoural regions of HCC tissues were included in the study. (A) The Pi content was detected by phosphomolybdic acid colorimetry. (B) and (C) Transcript levels of CD206, TGF‐β, iNOS and COX2 were determined by Q‐PCR, and correlation analysis with Pi content was performed. (D) Q‐PCR detected the distribution of CD206 and iNOS transcripts in peritumour and intratumour tissues. M2 markers: CD206 and TGF‐β; M1 markers: iNOS and COX2. Dots represent the mean expression value of each sample displayed as fold‐changes normalized to 1. Data are presented as the mean ± SEM. *p < 0.05, and n.s., no significance.

The detection results of HCC clinical samples showed that compared with the intratumour tissues, the peritumour tissues had a higher content of Pi (Figure 1A). Subsequently, these samples were used to determine the changes in phenotypic markers of macrophages. M2 macrophage markers included CD206, IL10, TGF‐β and CCL17, and M1 macrophage markers included iNOS and COX2. The results showed that the transcripts of CD206, TGF‐β, IL‐10 and CCL17 were positively correlated with the content of Pi in the peritumour tissues and intratumour tissues (Figure 1B and Figure S1A). Moreover, phenotypic analysis of M1 in patient samples showed that the content of Pi was negatively correlated with the expression of iNOS and COX2 (Figure 1C). This evidence indicates that in the tumour microenvironment, whether in the intratumour or the peritumour tissues, high levels of Pi are often accompanied by more M2 macrophages and fewer M1 macrophages, which reminds us that Pi content may affect the polarization of macrophages. Based on comprehensive analysis, we found that in these samples, there were more M2 macrophages and fewer M1 macrophages in the peritumour tissues than in the intratumour tissues. These results were obtained by analysing M2 and M1 phenotypic markers in transcripts (Figure 1D and Figure S1B).

### 3.2 Pi starvation leads to the repolarization of macrophages from M2 type to M1 type in an irreversible manner

Two polarization models, M1 and M2, were generated by PMA‐differentiated human THP‐1 monocytes (M0 macrophages) stimulated by LPS and IFN‐γ (M1‐polarized macrophages) or IL‐4 (M2‐polarized macrophages). CD68, a recognized marker of macrophages, was detected by Q‐PCR, and the results showed that M0 macrophages had marked upregulation of CD68 expression compared with that in THP‐1 cells, this means that the human monocytic THP‐1 cells can be successfully differentiated into macrophages (Figure S2A). After M0‐polarized macrophages were induced into M1‐ and M2‐polarized macrophages by adding different stimulators (M1: LPS + IFN‐γ; M2: IL‐4), M1 macrophages showed a spindle shape, while M2 macrophages showed a round shape (Figure S2B). Flow cytometry also showed that compared with M0 macrophages, M1 macrophages had more CD86‐positive cells, and M2 macrophages had more CD206‐positive cells (Figure S2C). In addition, the immunofluorescence experiment proved that the M1 and M2 polarization models were successfully constructed, which showed that compared with M0 macrophages, M1 macrophages had a higher level of iNOS expression, and M2 macrophages had a higher level of CD206 expression (Figure S2D). These results indicated that M0 macrophages could successfully differentiate into M1 or M2 macrophages under the stimulation of different factors.

**FIGURE 2:**
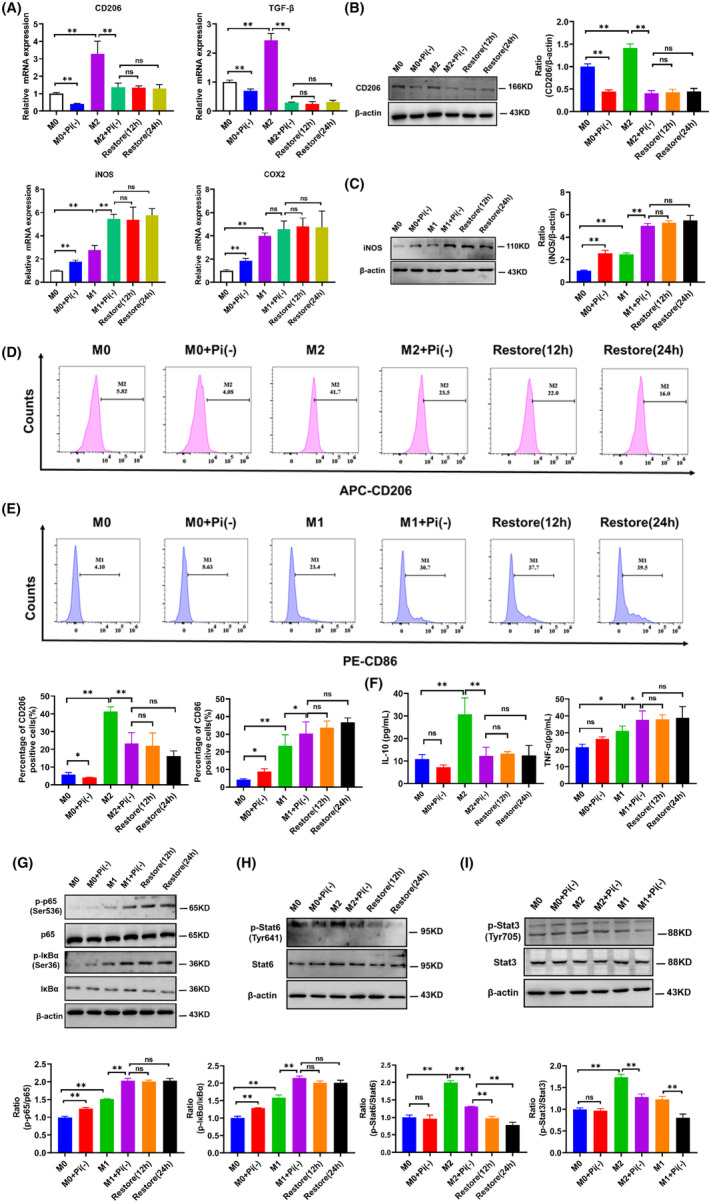
Pi starvation leads to the irreversible repolarization of macrophages from the M2 type to the M1 type. (A) Transcript levels of CD206, TGF‐β, iNOS and COX2 were determined by Q‐PCR. (B) and (C) The expression of CD206 and iNOS in macrophage cells was analysed by Western blotting after Pi starvation. (D) and (E) The percentages of CD206‐positive macrophages and CD86‐positive macrophages were determined by flow cytometry. (F) The cytokine levels of IL‐10 and TNF‐α were determined by ELISA. (G–I) The expression levels of p65, p‐p65, IκB‐α, p‐IκB‐α, Stat6, p‐Stat6, Stat3 and p‐Stat3 were analysed by Western blotting. Data are presented as the mean ± SEM from three independent experiments. *p < 0.05, **p < 0.01, and n.s, no significance.

To determine the effect of Pi starvation on the polarization of M2 macrophages, we first focused on the cytotoxicity of Pi starvation. As shown in Figure S2E, when macrophages were in the M0, M1 and M2 polarization states, deprivation of Pi in the culture medium produced cytotoxicity to macrophages, and no cytotoxicity to macrophages occurred when the concentration of Pi is equal to or higher than 31.25 mg/L. Therefore, we set the critical value of Pi concentration to 31.25 mg/mL to ensure that Pi starvation would not produce cytotoxicity in macrophages. Subsequently, we applied Pi starvation (31.25 mg/L) to M0 type, M2 type and M1 type macrophages and then applied Pi recovery treatment after Pi starvation. As shown in Figure 2A and Figure S2F, significant upregulation of M2 markers (CD206, TGF‐β, IL‐10 and CCL17) was observed after treatment with IL‐4 for 48 h, and the trend was greatly irreversibly reduced by Pi starvation, which can be obtained from M2 marker transcripts that remained unchanged after Pi recovery for 12 h and 24 h. Meanwhile, we found that M1 markers (iNOS, TNF‐α, CCR7 and COX2) were upregulated by treatment with Pi starvation in an irreversible manner. In addition, we observed that Pi starvation inhibited the expression of CD206 protein regardless of whether the macrophages were M0 or M2 type (Figure 2B) and promoted the expression of iNOS protein in M0 and M1 type (Figure 2C), which also showed the irreversibility of this effect. To further confirm the effect of Pi starvation on macrophage polarization, we measured the expression of CD206 and CD86 by flow cytometry in macrophages in different polarization states following treatment with Pi starvation. As shown in Figure 2D,E, M2 macrophages showed high expression of CD206 compared with M0 macrophages, and this trend was reduced by Pi starvation in an irreversible manner. While M1 macrophages displayed high CD86 expression levels, Pi starvation treatment irreversibly upregulated CD86 expression. In the detection of cell culture supernatant by ELISA, we found that the ratio of Th1/Th2 cytokines (Th1: TNF‐α; Th2: IL‐10) also indicated that Pi starvation inhibited M2 polarization and promoted M1 polarization (Figure 2F). Taken together, these results suggest that Pi starvation leads to repolarization of macrophages from the M2 type to the M1 type in an irreversible manner.

TLR4 pathway activation plays an important role in regulating M1 macrophage polarization. Western blot analysis showed that Pi starvation induced the expression of phosphorylated NF‐κB (p65) and phosphorylated IκB‐α (Figure 2G). In addition, immunofluorescence experiments also proved that Pi starvation can promote the nuclear transport of p65 (Figure S2G). Stat6 responds to IL‐4 stimulation and signal transduction, which is necessary for macrophage polarization into the M2 subtype. We then measured the protein level of Stat6, and the results showed that Pi starvation inhibited the p‐Stat6 level (Figure 2H). The subsequent immunofluorescence experiment also showed that Pi starvation inhibited the nuclear accumulation of Stat6 (Figure S2H). It is worth noting that the contributions of the Stat3 signalling pathway are complicated and function throughout the process of macrophage polarization, including M1 polarization and M2 polarization. Our evidence showed that in the M2 state, the phosphorylation of Stat3 was enhanced, while in the M1 state, Pi starvation inhibited the phosphorylation level of Stat3 (Figure 2I). Based on these results, we believe that Pi starvation reprogrammes macrophages by promoting the NF‐kB signalling pathway and inhibiting the stat3/stat6 signalling pathways.

### 3.3 Pi starvation‐mediated macrophage polarization inhibits the proliferation, migration and invasion of HCC cells

**FIGURE 3:**
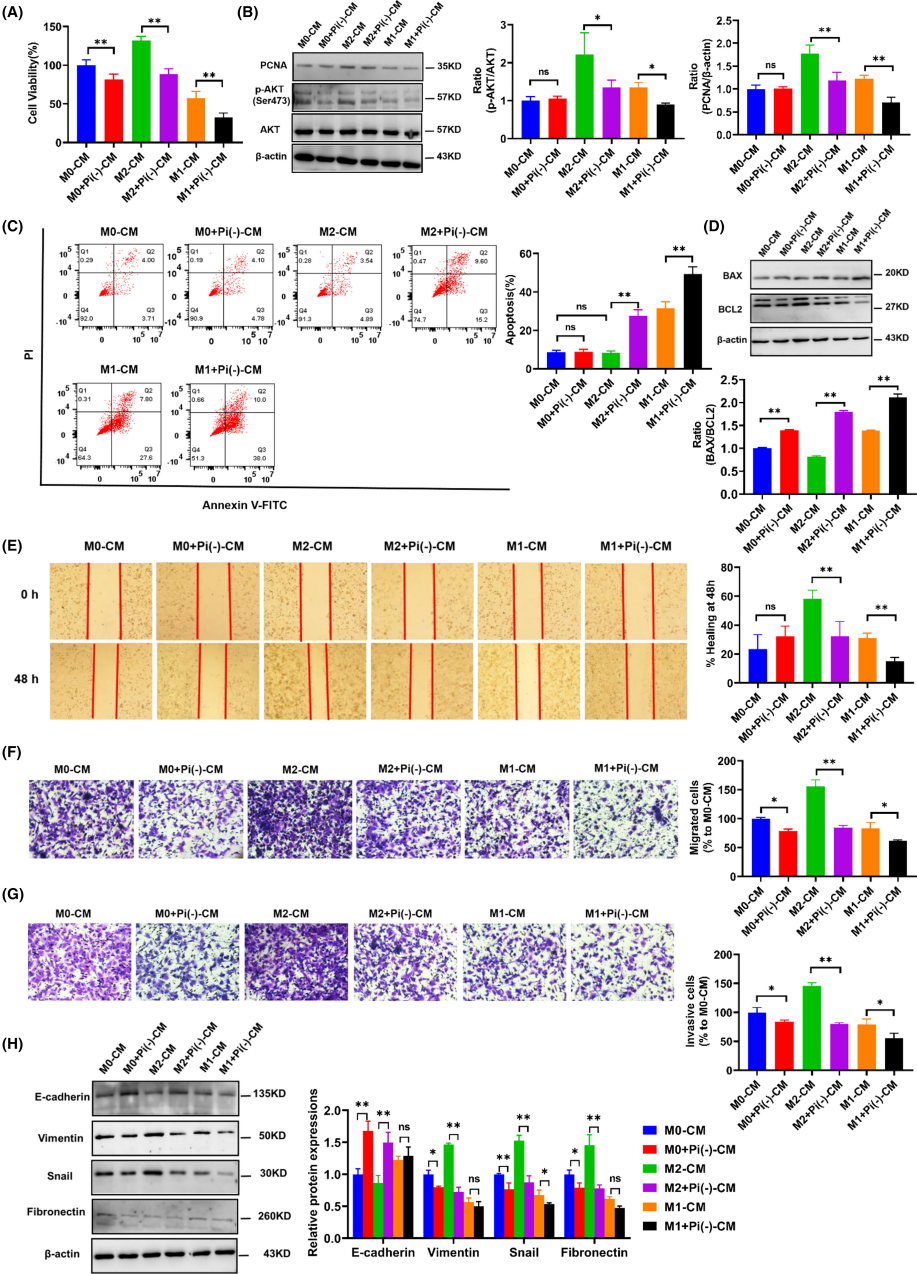
Pi starvation‐mediated macrophage polarization inhibited the proliferation, migration and invasion of HCC cells. The conditioned medium (CM) was collected from different macrophages, which were treated with or without Pi starvation. HepG‐2 cells were cultured with different CMs. (A) The viability of HepG‐2 cells was evaluated under various CMs for 48 h with MTT assays. (B) The expression of PCNA, AKT and p‐AKT in HepG‐2 cells was analysed by Western blot after treatment with various CM from macrophages. (C) The apoptosis of HepG‐2 cells treated with various CMs from macrophages was detected by flow cytometry. FITC, fluorescein isothiocyanate; PI, propidium iodide. (D) The expression of apoptosis‐related proteins (Bax and BCL‐2) was analysed by Western blotting after treatment with various CM from macrophages. The effect of CM on HepG‐2 cell migration was evaluated by wound scratch assay (E) and transwell assay (F). (G) The invasion of HepG‐2 cells was analysed in a transwell system. (H) The expression levels of EMT‐related markers, including E‐cadherin, fibronectin, vimentin and snail, were examined by Western blotting in HepG‐2 cells treated with CM from macrophages. Data are presented as the mean ± SEM from three independent experiments. *p < 0.05; **p < 0.01, and n.s., no significance.

To further verify whether Pi starvation‐mediated macrophage polarization directly influences the proliferation of cancer cells, we observed that M2‐CM promoted the proliferation of HepG‐2 and SMCC‐7721 cells, but the promotion effect was inhibited by Pi starvation. By contrast, M1‐CM inhibited the proliferation of HepG‐2 and SMCC‐7721 cells, and this inhibitory effect was also amplified by Pi starvation (Figure 3A and Figure S3A). Similar results regarding the proliferative ability of HepG‐2 cells with diverse treatments were obtained when examining PCNA expression by Western blot (Figure 3B). As expected, the results of flow cytometry analysis revealed that Pi starvation in both M1 and M2 states significantly increased apoptosis of HepG‐2 cells induced by the macrophage‐conditioned medium. (Figure 3C). In addition, the expression levels of apoptosis‐related proteins, including Bax and BCL‐2, were detected in HepG‐2 cells treated with conditioned medium. We found that M2‐CM can inhibit the apoptosis of HepG‐2 cells, while M1‐CM promotes apoptosis, and the apoptosis of these macrophages on HepG‐2 cells was aggravated by Pi starvation (Figure 3D). These results indicated that the Pi starvation medium can not only promote the apoptosis of cancer cells but also inhibit their proliferation.

Given the key role of cytokines in cell–cell interactions, we sought to determine whether Pi starvation‐mediated macrophage polarization affects cancer cells. M2‐CM significantly promoted the migration of HepG‐2 and SMCC‐7721 cells in wound‐healing assays, whereas M2‐CM from combined treatment with Pi starvation significantly reduced this effect. M1‐CM significantly inhibited the migration of HepG‐2 and SMCC‐7721 cells, and M1‐CM combined with Pi starvation promoted this effect (Figure 3E and Figure S3B). In addition, transwell assays were used to determine the migration and invasion ability of HepG‐2 and SMCC‐7721 cells. Compared with M0‐CM culture, M2‐CM promoted the migration and invasion of cancer cells, contrary to M1‐CM; however, M2‐CM or M1‐CM combined with Pi starvation treatment inhibited the migration and invasion ability of HepG‐2 and SMCC‐7721 cells (Figure 3F,G, Figure S3C,D). EMT process refers to the process in which epithelial cells undergo a transformation towards a mesenchymal phenotype, characterized by loss of polarity and cell‐to‐cell adhesion, and acquisition of migratory and invasive properties similar to mesenchymal cells, it is highly significant for the metastasis of HCC. Subsequently, Western blotting was performed to analyse the EMT markers (E‐cadherin, vimentin, snail and fibronectin) in HepG‐2 cells after culture with macrophage medium. As shown in Figure 3H, the expression of the epithelial marker E‐cadherin was reduced, while the mesenchymal markers vimentin, snail and fibronectin were upregulated. Considering all these results, we believe that Pi starvation‐mediated macrophage polarization inhibits the migration and invasion of cancer cells by EMT process.

### 3.4 In vivo therapeutic validation of Pi starvation

**FIGURE 4:**
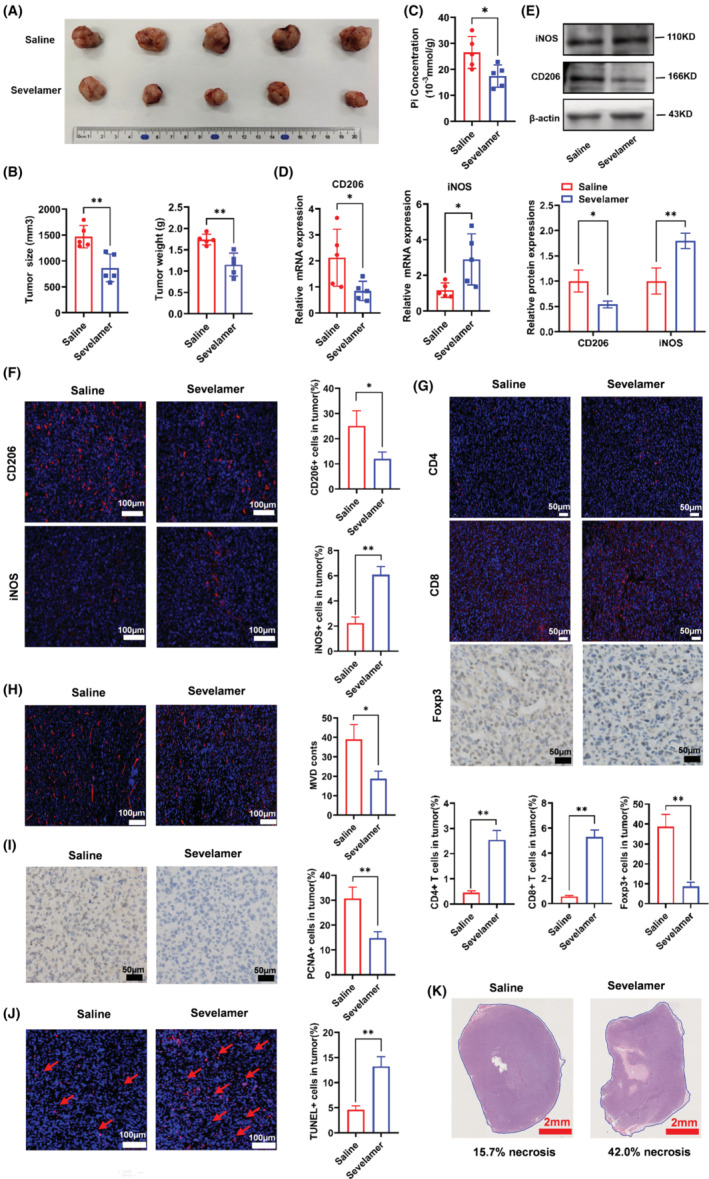
In vivo therapeutic validation of low‐Pi stress. (A) Tumour image after 14 days of intratumoural injection or noninjection of sevelamer. (B) The tumour size and weight were measured. (C) The Pi content of tissues. (D) Transcript expression levels of macrophage markers in tumour tissues of each group. All expression levels were normalized to the mean expression of the saline group. (E) The protein levels of macrophage markers in tumour tissues of each group. (F) The infiltrated macrophages in tumour tissues were stained with CD206 and iNOS by immunofluorescence. (G) The infiltrated Tregs, CD4+ T cells and CD8 + T cells in tumour tissues were stained with Foxp3, CD4 and CD8 by IHC and immunofluorescence. (H) The levels of CD34 were measured by immunofluorescence to assess tumour vessel maturation. (I) The levels of PCNA were measured by IHC to assess tumour proliferative activity. (J) Apoptosis was evaluated by TUNEL assay. (K) Representative histological haematoxylin and eosin staining of tumour lesions 14 days after treatment, average necrosis was presented in the below the image. M2 marker: CD206; M1 marker: iNOS. *N* = 5 animals per group. *p < 0.05; **p < 0.01 and n.s, no significance.

The subcutaneous mouse model was established by the subcutaneous injection of Hep1‐6 cells. We first evaluated the therapeutic effect of intratumoural injection of sevelamer, which resulted in significant decreases in either tumour weight or volume compared with those in the control cohort of mice (Figure 4A,B), this indicates that sevelamer can significantly inhibit tumour growth. Accordingly, we then examined whether intratumoural injection of sevelamer would change the content of Pi in the tumour. As shown in Figure 4C, intratumoural injection of sevelamer significantly reduced the content of Pi in the tumour. This is also consistent with the function of sevelamer in combining inorganic phosphorus, moreover, the inhibitory effect on tumours is likely to be caused by Pi deprivation. Subsequently, we examined whether Pi starvation could re‐educate TAMs to an antitumour M1‐like phenotype. Q‐PCR results showed that the sevelamer injection array had a higher iNOS transcript level and a lower CD206 transcript level (Figure 4D). In addition, the changes in iNOS and CD206 protein expression were confirmed by immunofluorescence and Western blotting. As shown in Figure 4E,F, CD206 expression in tumour tissues was reduced in the sevelamer treatment group, while iNOS was enhanced. Together, these results suggested that Pi starvation treatment exerts its antitumour effect by inhibiting M2 polarization and promoting M1 polarization.

Activation of adaptive immunity is an important direction for innate immunity to function. Here, we detected three adaptive immune indicators in the tumour region, including CD4+ T, CD8+ T and Tregs cells. The immunofluorescence results showed that the intratumoural injection of sevelamer significantly promoted the infiltration of CD4+ T and CD8+ T cells in the tumour region, which promoted the effect of immune promotion. Simultaneously, IHC results showed that intratumoural injection of sevelamer inhibited the expression of Foxp3, which was a sign of Treg cell reduction (Figure 4G). Because tumour angiogenesis contributes to tumour metastasis, we then analysed the effect of intratumoural injection of sevelamer on vessel density. As shown in Figure 4H, microvessel density (MVD), as indicated by CD34 staining, was significantly reduced by sevelamer treatment, this demonstrates a certain antitumour angiogenesis effect. To study whether sevelamer treatment is related to the imbalance between cell proliferation and apoptosis. Cell proliferation was estimated by PCNA immunohistochemical staining, and apoptosis was determined by a TUNEL assay. Our results showed that the number of PCNA positive cells was significantly reduced, and the percentage of TUNEL positive cells was significantly increased after the treatment of sevelamer (Figure 4I,J). Tumour necrosis outcomes were compared 14 days after the treatment of sevelamer. The necrosis ratio in the sevelamer administered groups was significantly higher than that in the saline administered, indicating a better therapeutic effect (Figure 4K).

## 4 DISCUSSION

**FIGURE 5:**
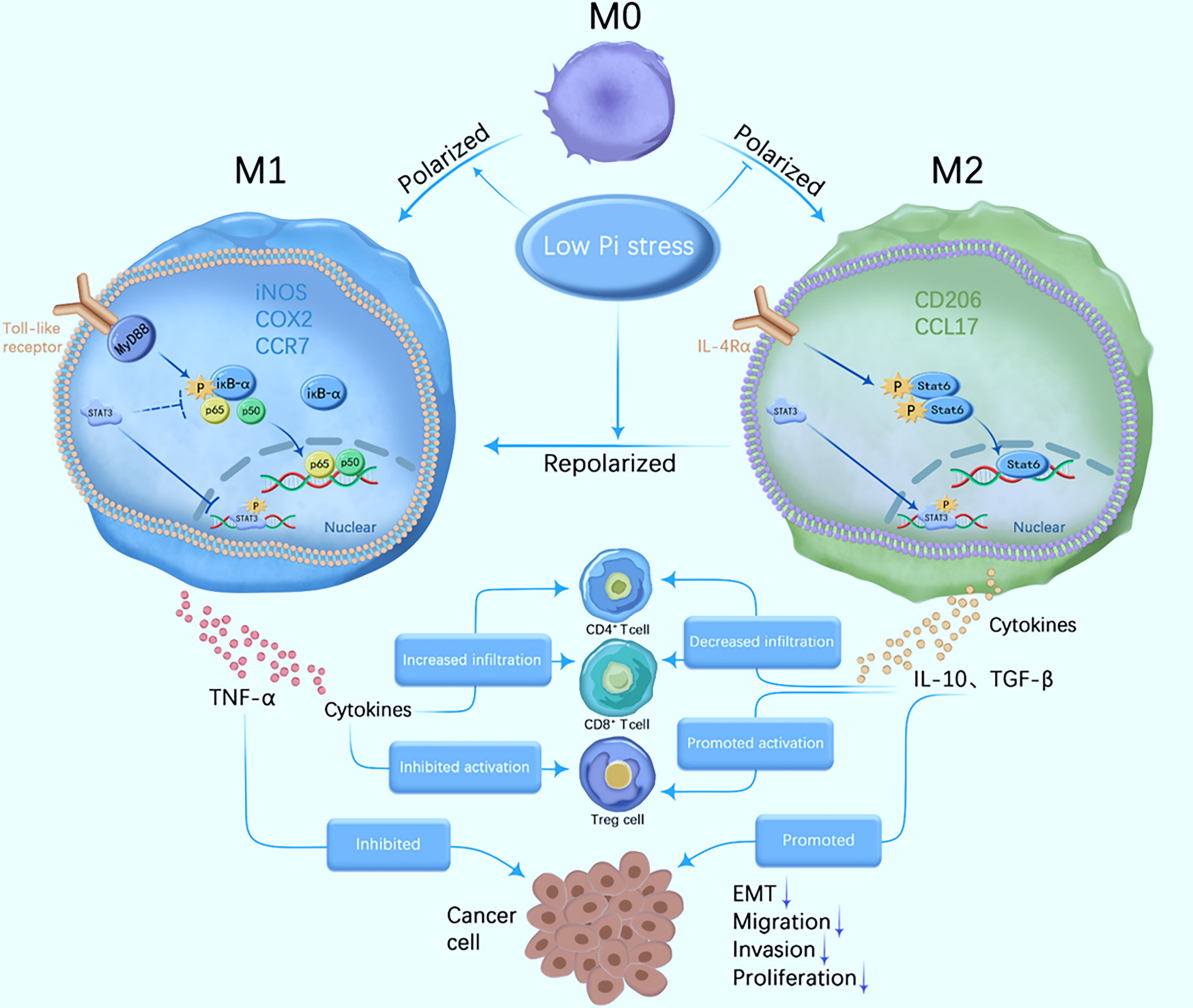
The mechanism of Pi stress‐mediated tumour inhibition. Pi stress blocks the Stat3 and Stat6 signalling pathways of macrophages and activates the NF‐кB signalling pathway of macrophages, together reprogramming TAMs to the M1 phenotype and increasing CD4+ T and CD8+ T‐cell infiltration. Low‐Pi stress simultaneously inhibits Treg cells, contributing to CD4+ T and CD8+ T‐cell activation and tumour suppression.

Pi is essential for many, if not all, living organisms because of its roles in several biochemical processes, such as kinase/phosphatase signalling; ATP formation; and lipid, carbohydrate and nucleic acid biosynthesis.^27^ Our previous studies have shown that low‐Pi stress inhibits the progression of HCC in vitro and in vivo.^28^ However, it is not clear how low‐Pi stress inhibits cancer cell growth. Cancer cells partially lose the ability to migrate and invade, but cell proliferation is still active, which is inconsistent with the in vivo anticancer test; thus, we thought that low‐Pi stress might regulate the inflammatory microenvironment on immune cells and then indirectly kill cancer cells. Herein, we initiated this study by analysing the correlation between the intratumoural and peritumoural Pi concentrations of two polarized phenotypes of macrophages from HCC patient specimens, and more M1 macrophages than M2 macrophages were observed in lesions with low‐Pi concentrations. An in vitro test revealed that the polarization of THP‐1 monocytes was modulated by this new stress factor. Low‐Pi stress silences the expression of stat6 and activates the expression of p65, thus irreversibly stabilizing the M1 phenotype. The conditioned medium, containing cytokines released by THP‐1 cells under low‐Pi stress, showed an anticancer effect on the progression of HepG‐2 and SMCC‐7721 cell lines via the inhibition of EMT. In a subcutaneous liver cancer model, phosphate binder was intratumourally injected to decrease the tumoural Pi, which drove the tumour microenvironment to become more immunostimulatory as the M1 phenotype population increased and more immune cells (CD8 + &CD4+ T cells) infiltrated, whereas the number of Treg cells decreased. Therefore, low‐Pi stress induced macrophage polarization, promoting both the innate and adaptive anticancer immune responses, and the whole process is shown schematically in Figure 5.

Generally, tumours recruit both circulating monocytes and tissue‐resident macrophages to the TME and polarize them towards an M2 phenotype, creating TAMs, via a variety of soluble and mechanical factors. TAMs enhance tumour progression by promoting genetic instability, angiogenesis, fibrosis, immunosuppression, lymphocyte exclusion, invasion and metastasis. Hepatocellular carcinoma (HCC) progresses in an immunosuppressive microenvironment, and alternatively activated (M2) macrophages, as the dominant form of TAM, promote growth and invasiveness in solid tumours. The clinical association between the tumour‐promoting effects of M2 macrophages and adverse survival outcomes has been widely described,^29^, ^30^ and the M2 phenotype has prognostic value for predicting survival. More precisely, the number of M2 macrophages in the peritumoural region is higher than that in the intratumour region.^15^ By contrast, M1 macrophages play a key role in host antitumour defences in HCC, clinically expecting longer survival.^16^, ^31^ Concurrently, we found that the population of peritumoural macrophages was higher than that within the tumours. More importantly, the populations of M2 and M1 macrophages were linearly correlated with Pi concentration. Therefore, intratumoural Pi might be a new prognostic factor for HCC. Although the causal relationship between Pi and the polarization of TAMs is unclear, we propose the hypothesis that low‐Pi stress could repolarize TAMs into the M1 phenotype, inhibiting HCC progression and leading to a better prognosis.

Pi starvation could inhibit the polarization of M2 macrophages and promote the polarization of M1 macrophages, as was proven in vitro. The IL‐6/STAT3 pathway mediates M1/M2 macrophage polarization during the development of HCC, and inhibition of the STAT3 signalling pathway can induce M1 macrophage polarization and inhibit M2 macrophage polarization.^32^ In this study, we demonstrated that Pi starvation inhibited phosphorylated STAT3. Furthermore, NF‐кB, another key transcription factor related to macrophage M1 activation, regulates the expression of a large number of inflammatory genes. Proteome degradation of IкB and the release of the p65/p50 dimer is an important process of NF‐кB signal activation.^33^, ^34^, ^35^ Our observations revealed that Pi starvation promotes the phosphorylation of p65 and IкBα, and the increased nuclear accumulation of p65 activates the NF‐кB pathway. Additionally, the activation of the STAT6 signal activator increased the secretion of IL‐4, promoted differentiation into M2 myeloid cells and inhibited M1 polarization.^36^ IL‐4 and IL‐13 are important M2‐polarized cytokines that act through STAT6 by inducing STAT6 phosphorylation and promoting the transcription of STAT6 response genes.^37^ We found that Pi starvation inhibited the phosphorylation level of STAT6 in the M0 phenotype and M2 phenotype. The increase in immunofluorescence proved that Pi starvation inhibited the nuclear transport of STAT6. Therefore, at least three polarization‐relevant pathways are modulated under low‐Pi stress.

TAM repolarization, induced by low‐Pi stress, suppressed tumour cell metastasis and invasion by releasing cytokines and inhibiting the EMT process. Emerging evidence has clearly shown that M2 macrophages promote the EMT process of HCC cells by secreting cytokines, while M1 macrophages play the opposite role.^38^ Remarkably, our research has suggested a new strategy in which Pi starvation inhibits macrophage‐mediated migration and invasion of HCC cells. The expression levels of N‐cadherin, snail, vimentin and fibronectin were all significantly upregulated in HCC cells cultured in macrophage conditional medium with Pi starvation. By contrast, the expression level of E‐cadherin was greatly reduced. As previously reported,^39^ variation in those biomarkers representing EMT was greatly inhibited under low‐Pi stress. In addition to EMT‐mediated metastasis and invasion, cancer cells have weak proliferation ability in medium containing the released cytokines, and low levels of PCNA expression and p‐AKT are observed, indicating that Pi starvation‐mediated macrophage‐conditioned medium promotes the apoptosis of HCC cells due to the activation of PI3K/AKT signal transduction.^40^

In addition to the direct killing of tumour cells, polarized macrophages also act as antigen‐presenting cells in adaptive immunity, as we proved in the animal model. It is well known that tumour cells escape immune surveillance because TAMs (M2) have poor antigen presentation ability and inhibit the immune response of T cells by releasing immunosuppressive factors.^41^ Recently, some studies on TAM‐targeted cancer therapy have focused on the following strategies: inhibiting the recruitment of macrophages, transforming tumour‐promoting M2 macrophages into antitumour M1 macrophages and inhibiting the survival of TAMs.^42^ Low‐Pi stress repolarized TAMs into the dominant M1 phenotype and modulated T cells and DCs, as shown in Figure 5. Generally, macrophages are affected by tumour cells after entering the TME and enter a polarization state in which they promote tumour growth and inhibit T‐cell function. Therefore, TAMs are one of the leading factors inducing T‐cell dysfunction. CD8+ T cells have direct cytotoxicity to target cells and play a crucial role in antitumour therapy. When immature CD8+ T cells undergo differentiation, they can form mature effector CD8+ T cells, which can effectively eliminate antigens.^43^ Previous studies have shown that macrophages can also express inhibitory receptor PD‐1, cytotoxic T‐lymphocyte antigen 4 (CTLA‐4), T‐cell immunoglobulin and mucin containing molecule 3 (Tim‐3); these inhibitory ligands induce T‐cell apoptosis or functional inactivation.^44^, ^45^, ^46^ CD4+ T cells are stimulated to proliferate and differentiate into the TH1 table by recognizing antigens processed by dendritic cells (DCs) and/or exposed to DC‐derived IFN‐I and IL‐12.^47^ Emerging evidence suggests that CD4+ TH1 cells are stimulatory immune cells that are mainly used as auxiliary regulators to enhance the antitumour immunity of CD8+ T cells. The metastasis of CD4+ T cells greatly enhanced the antitumour response of CD8+ T cells. Compared with CD8+ T cells, CD4+ T cells easily infiltrate and proliferate in tumour tissue, highlighting their importance in antitumour immunity.^48^ Regulatory T (Treg) cells are immunosuppressive subsets of CD4+ T cells. They express inhibitory receptors CD25, CTLA‐4 and TIM‐3 on the cell surface, especially the typical transcription factor Foxp3. There is evidence that Foxp3+ Treg cells increase in the peripheral blood of HCC patients and infiltrate into tumours, becoming an independent prognostic factor for overall survival.^49^ Similar to TAMs, Treg cells can promote the proliferation of tumour cells and promote tumour development by promoting T‐cell depletion and reducing the infiltration of stimulating immune cells.^50^

Our study has several limitations. Firstly, sevelamer, being an insoluble polymer instead of a small molecule, does not spread and diffuse after intratumoural injection. This lack of diffusion does not disturb the electrolyte balance in the peripheral circulation. However, clinical intratumoural Pi deprivation is only feasible through local‐regional techniques such as transcatheter arterial embolization for HCC therapy. This technique may increase the level of risk associated with administration. Secondly, the exact mechanism of irreversible polarization is still unclear. It cannot be ruled out that the intratumoural injection of sevelamer itself induced tumour necrosis, thereby triggering a proinflammatory response and immune cell infiltration. In other words, the necrotic effect may not be mediated through macrophage polarization. Lastly, this work only reveals a small portion of the overall picture, as tumour biology under low‐Pi stress may be more complex than expected. Additionally, our results may be biassed since we assume that high‐Pi stress drives tumour progression, whereas the true causation is still unknown and could even be in the opposite direction.

## 5 CONCLUSIONS

In summary, patient specimen analysis indicates that intratumoural Pi is a chemical marker associated with TAM polarization and the immune microenvironment. Preclinical studies have revealed that low‐Pi stress induces repolarization of M2 macrophages to M1 macrophages, and the re‐education of this phenotype will inhibit the proliferation, migration and invasion of HCC cells. Moreover, low‐Pi stress enhanced antitumour immunity and halted tumour progression by promoting the infiltration of CD4+ & CD8 + T cells, and inhibiting the infiltration of Tregs in murine models. Pi starvation modulates the cancer immune microenvironment as a prospective monotherapy or combined treatment of HCC.

## AUTHOR CONTRIBUTIONS

**Yang‐feng Lv:** Conceptualization (lead); data curation (lead); formal analysis (lead); investigation (lead); methodology (equal); resources (equal); software (supporting); validation (equal); visualization (equal); writing – original draft (lead). **Zi‐qiang liao:** Data curation (equal); formal analysis (equal); investigation (supporting); methodology (supporting); resources (supporting); validation (supporting); visualization (supporting). **Qiu‐chen Bi:** Data curation (equal); formal analysis (equal); investigation (supporting); methodology (supporting); validation (equal). **Chuan‐sheng Xie:** Data curation (supporting); formal analysis (supporting); investigation (supporting); methodology (equal); software (equal); validation (equal); visualization (equal). **Xiao‐yong Wei:** Conceptualization (supporting); formal analysis (supporting); investigation (supporting); supervision (supporting). **Yi Yun:** Resources (equal); validation (equal). **Yuan‐qiao He:** Conceptualization (supporting); formal analysis (supporting); resources (supporting). **Qun Tang:** Conceptualization (lead); funding acquisition (lead); project administration (lead); writing – review and editing (lead).

## FUNDING INFORMATION

This work was financially supported by the National Natural Science Foundation of China (82060335) and the Jiangxi Provincial Natural Science Foundation (20212ACB206037).

## CONFLICT OF INTEREST STATEMENT

The authors confirm that there are no conflicts of interest.

## Supporting information


Figure S1

Figure S2

Figure S3

## Data Availability

The datasets generated during and/or analyzed during the current study are available from the corresponding author upon reasonable request.
