# Supplementary figures and images for: Irreversible repolarization of tumour‐associated macrophages by low‐Pi stress inhibits the progression of hepatocellular carcinoma
Source: J Cell Mol Med. 2023 Jul 20;27(19):2906–21. doi: 10.1111/jcmm.17861 (PMC10538272; doi:10.1111/jcmm.17861)

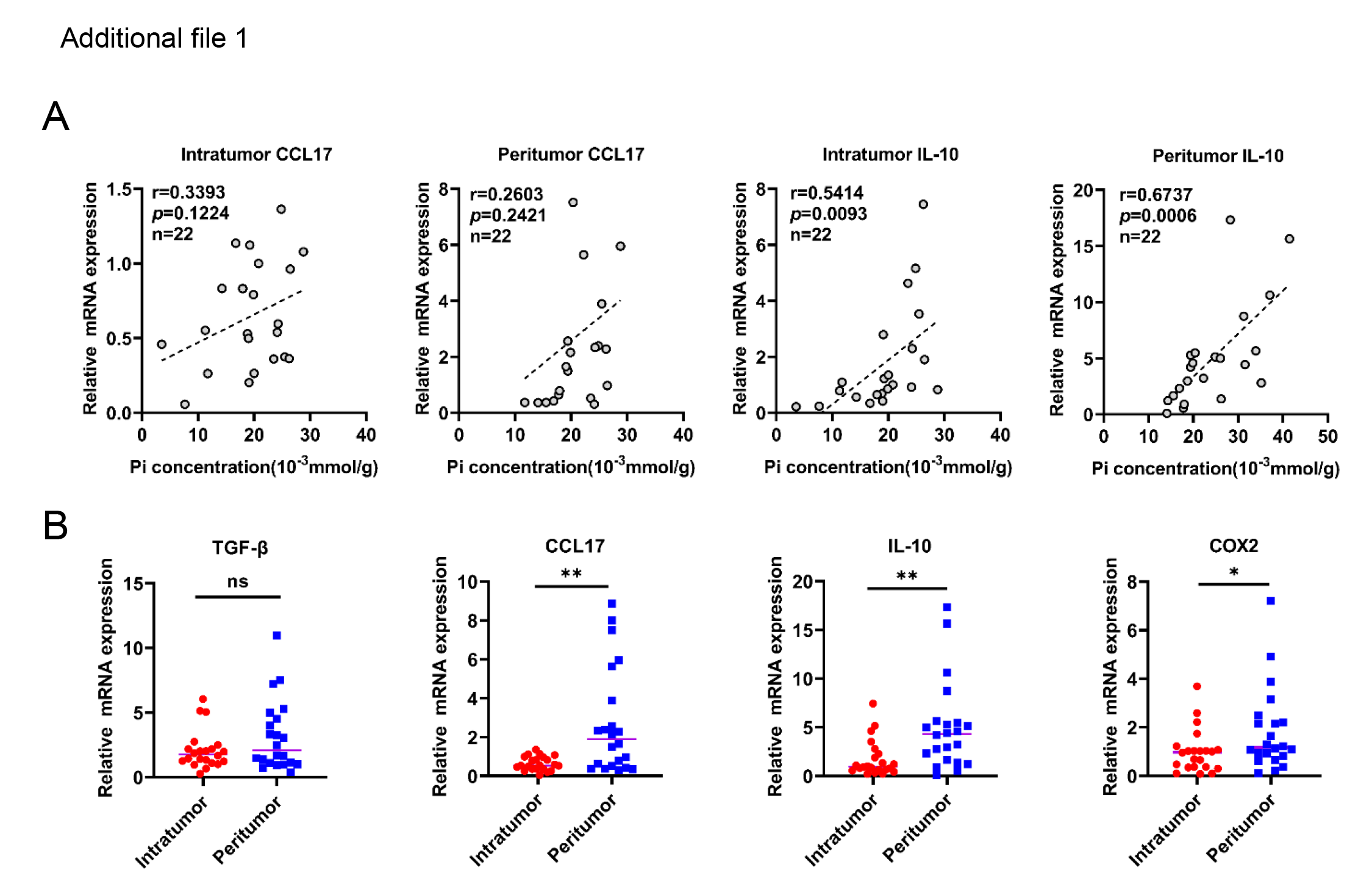

Supplement: Supplementary file 1 — Figure S1 [file JCMM-27-2906-s003.tif]

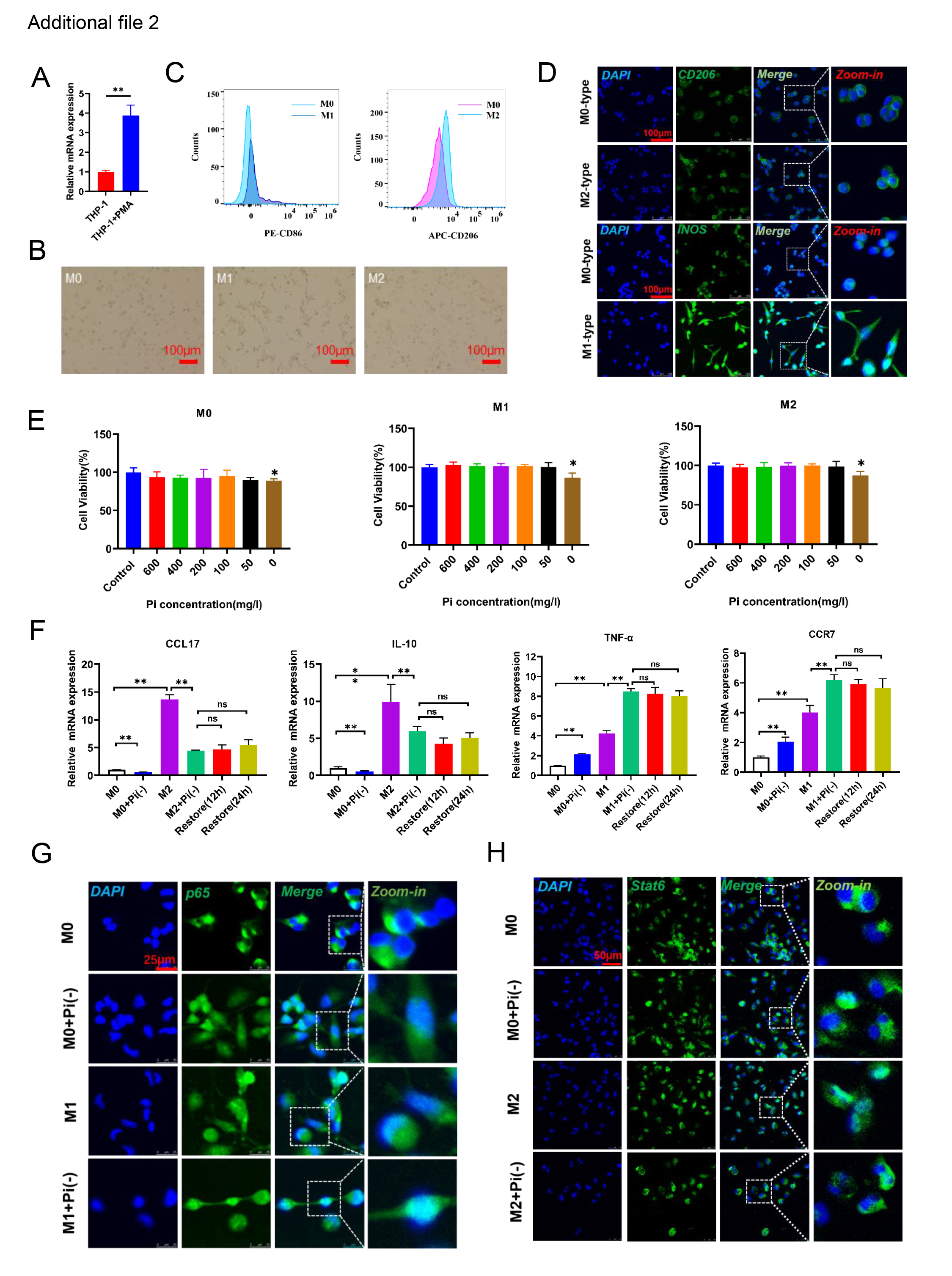

Supplement: Supplementary file 2 — Figure S2 [file JCMM-27-2906-s001.tif]

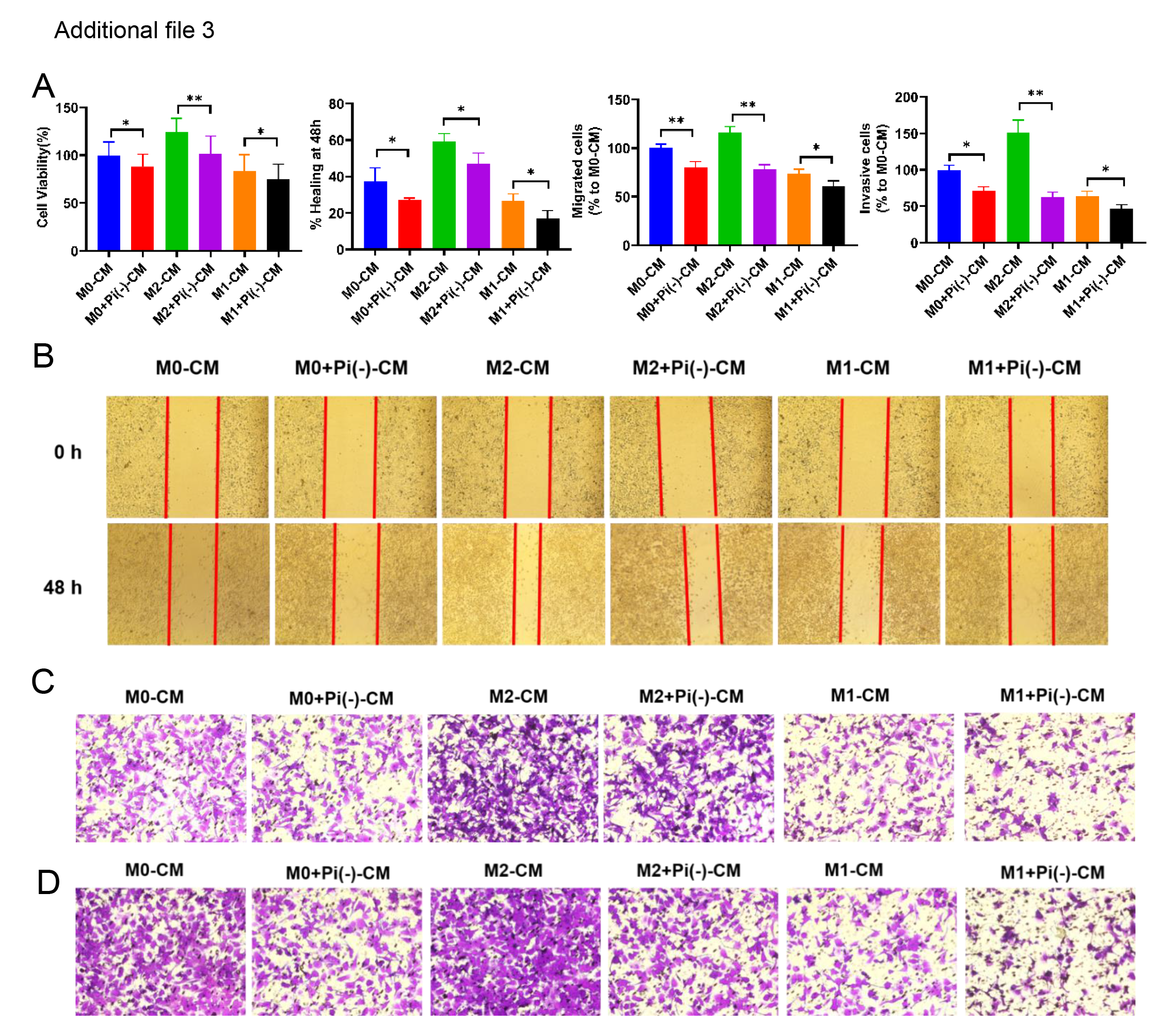

Supplement: Supplementary file 3 — Figure S3 [file JCMM-27-2906-s002.tif]
